# Mapping assessment practices in DanceSport: a Human-In-The-Loop systematic review

**DOI:** 10.3389/fpsyg.2026.1847089

**Published:** 2026-07-22

**Authors:** Yuwei Zhu, Guangyuan Liu, Xiaoyu Wang

**Affiliations:** 1 Arts College, Northeastern University of China, Shenyang, Liaoning, China; 2 Nanjing Sports Dance Association, Nanjing, Jiangsu, China

**Keywords:** DanceSport, Human-In-The-Loop, LDA topic modeling, performance assessment, systematic review

## Abstract

**Background:**

DanceSport is an aesthetic sport that combines athletic execution, artistic expression, musicality, and partner interaction. Although formal adjudication remains central to DanceSport competition, recent studies have increasingly explored technology-assisted approaches to provide more measurable and traceable evidence. However, the field lacks a focused synthesis explaining what major themes have emerged, how these themes have developed, what technologies have been applied, and how existing systems have been validated.

**Methods:**

This study conducted a systematic review enhanced by Human-In-The-Loop topic modeling. Following PRISMA-informed procedures, 1,957 records were retrieved from the Web of Science Core Collection, 192 full-text articles were assessed for eligibility, and 24 studies were included in the final review. Exploratory LDA topic modeling was applied to study abstracts to identify major themes and similarity-based thematic transitions, while qualitative content analysis of full texts examined assessment approaches, technologies, dance-category coverage, and validation designs.

**Results:**

Four major themes were identified: 3D dance motion perception, dance posture and motion evaluation, image analysis of dynamic dance performance, and validation of dance evaluation. Together, these themes reveal a research landscape that is increasingly centered on objective and measurable performance evidence. Building on earlier concerns with judging validity, the field has gradually shifted toward technology-assisted and data-driven assessment, with research focusing primarily on indicators such as posture, motion, balance, rhythm, and movement recognition. To support this transition, studies have increasingly employed motion capture, wearable sensors, computer vision, and computational models. Nevertheless, despite these technological advances, validation remains limited by uneven dance-category coverage and a continued reliance on isolated movements, short performance clips, or laboratory-based tasks.

**Conclusions:**

DanceSport assessment research is moving toward technology-assisted and multimodal evidence systems, but technical measurement has not yet been fully integrated with aesthetic judgment, expert scoring, and competition-level validation. Future research should develop cross-dance datasets, compare Standard and Latin dances, test complete competition routines, examine criterion validity between technical indicators and expert scores, and involve judges, coaches, and athletes in assessment frameworks.

## 1 Introduction

DanceSport, a competitive form of ballroom dance, is commonly organized into two main categories with 10 recognized competition dances: International Standard Dance, including Waltz, Viennese Waltz, Foxtrot, Quickstep, and Tango, and International Latin Dance, including Rumba, Cha Cha, Jive, Samba, and Paso Doble (Dai et al., 2024; Liu et al., 2024). As a representative aesthetic sport, DanceSport provides a distinctive intersection between athletic performance and artistic expression, where aesthetic experience contributes pragmatic value to the development of sport (Hopsicker, 2015; Markula, 2018). Like other aesthetic sports, such as gymnastics and figure skating, DanceSport requires performers to pursue competitive effectiveness through technically controlled movement while maintaining aesthetic quality as an integral condition of performance excellence (Markula, 2018).

This dual demand makes standardized assessment central to the institutional development of DanceSport. Competitive outcomes not only determine dancers' success (Hopsicker, 2015, p. 214), but also shape training, education, governance, visibility, and public legitimacy (Picart, 2006, p. 84). Therefore, recent discussions have highlighted the challenge of assessing dance performance in ways that are both rigorous and publicly legitimate (Italian DanceSport and Musical Sports Federation (FIDESM), 2024; Ruscello et al., 2024). Compared with many competitive sports, DanceSport assessment is more subjective and multidimensional. It involves not only measurable technical qualities, such as accuracy and control, but also aesthetic judgments related to interpretation, expression, presentation, and appearance (Geukes et al., 2023). Debates surrounding *breaking* within the World DanceSport Federation (WDSF) illustrate this tension: while sportification can enhance visibility and institutional recognition, it may also risk subordinating aesthetic distinctiveness to standardized athletic criteria (Li and Vexler, 2019). However, Figure skating offers a relevant comparative case among aesthetic sports. The International Skating Union (ISU) judging system separates technical elements from program components and supports technical scoring through video replay, demonstrating how technical objectivity and aesthetic evaluation can be integrated within a standardized framework (The International Skating Union, 2026). Its institutional legitimacy provides a valuable reference for understanding how aesthetic sports can balance competitive credibility with artistic values.

Since the early twentieth century, DanceSport competitions have relied on judge-based adjudication, and their assessment systems have evolved through the interaction of organizations that promoted different visions of competitive ballroom dance (Markula, 2018). Within contemporary DanceSport governance, the WDSF has played a central role in codifying competition rules and adjudication principles. WDSF-based technical documents indicate that International Standard Dance and International Latin Dance differ substantially in movement structure, body posture, rhythmic organization, and partner relationship, although they are often evaluated through a broadly shared set of adjudicative criteria. Table 1 summarizes these differences and shows why DanceSport assessment cannot be reduced to a single generic scoring model.

**Table 1:** WDSF-based comparison of technical and adjudicative characteristics in International Standard and Latin Dance^a^.

Dimension	International standard dance	International Latin dance
Movement structure	Continuous couple movement; closed-position structure; formalized traveling patterns	Dance-specific character; expressive actions; intensity and energy
Body posture	Closed position; maintained hold; formal couple frame	More expressive body use; character-based styling; freer visual presentation
Rhythmic characteristics	Music-led continuity; controlled timing; dance-specific formal rhythm	Accented rhythm; energetic timing; expressive musicality
Partner relationship	Stable partner connection; continuous hold; unified couple frame	Variable partner interaction; expressive connection; character-driven partnership
Judging criteria	Posture; dance holds; center; balance; foot skills (foot action and foot placement); body actions; drive actions; preparation to move; rise and fall; swing; pivots/pivoting actions/continuous spins; skilled figures	

^a^Information cites from the official website of the World DanceSport Federation (WDSF): https://www.worlddancesport.org/.

At present, the mainstream evaluation framework in DanceSport still relies largely on expert adjudication (Monleon et al., 2018). Judges must rapidly evaluate technical execution and artistic presentation based on perception and experience (Premelč et al., 2019). However, such systems remain vulnerable to rater effects, differences in expertise, and inconsistencies in judgment (Amoruso et al., 2014; Parke et al., 2006; Sevdalis and Raab, 2016). Prior studies have shown that adjudicators' physical, emotional, and cognitive states may affect decision quality (Monleon et al., 2018), and that familiarity with movement vocabulary can also shape evaluative judgments (Calvo-Merino et al., 2010). These findings reinforce a central challenge in DanceSport research: how to develop assessment systems that reduce bias while preserving sensitivity to performance quality.

In response, researchers have increasingly explored more objective and technology-assisted approaches to DanceSport evaluation. Existing work spans scoring criteria, fairness of judgment, and the design of performance evaluation systems, often drawing on interdisciplinary methods and cross-modal evidence (Anderlucci et al., 2021; Tsuchida et al., 2019). Biomechanical and motion-based studies are particularly important because they provide measurable evidence for modeling, analyzing, and comparing dance movement patterns (Oliveira et al., 2012; Wang and Zong, 2023). These developments suggest an emerging shift from purely subjective judging toward technology-assisted and data-driven assessment, yet existing methods remain fragmented and have not been integrated into a widely accepted competitive assessment framework.

Given this fragmentation, reviewing DanceSport assessment research is both necessary and timely. Existing reviews have examined broader applications of movement analysis (Aritzeta et al., 2022; Quadrado et al., 2022), but the field still lacks a focused synthesis despite its wide participation and growing methodological diversity (Badiola-Bengoa and Mendez-Zorrilla, 2021). A conventional narrative review would struggle to capture this scattered and interdisciplinary literature in a transparent and systematic way. Advances in natural language processing and statistical text mining have made it possible to extract latent thematic structures from heterogeneous bodies of literature, thereby reducing the limitations of purely manual synthesis while preserving the need for expert interpretation (Kee et al., 2019). Moreover, research has shown that systematic reviews employing Human-In-The-Loop (HITL) framework demonstrate greater reliability and adaptability than traditional bibliometric software approaches (Anesbury and Stocchi, 2025). For that reason, this study adopts a systematic review enhanced by HITL framework, combining the scalability of latent dirichlet allocation (LDA) topic modeling with researcher-led interpretation. This design makes it possible to map the thematic structure of the literature, identify major thematic patterns and similarity-based thematic transitions, and then connect those quantitative patterns to a qualitative synthesis of evaluation systems, assessment targets, technologies, and experimental designs. Through this combined strategy, the review clarifies how DanceSport assessment research has evolved, where its methodological gaps remain, and which directions are most promising for building standardized, interpretable, and comparable assessment frameworks.

To address these gaps, this study conducts a systematic review enhanced by Human-In-The-Loop topic modeling to provide a systematic and methodologically transparent synthesis of DanceSport assessment research. Specifically, the review is guided by four research questions: (1) What major research themes have emerged in DanceSport assessment research? (2) How have these themes developed over time, and do they indicate a shift similar to the assessment paradigm changes observed in other aesthetic sports? (3) What technologies and methodological approaches have been applied to DanceSport assessment? and (4) How have these approaches been validated in existing studies?

In doing so, this study does not merely summarize existing literature, but also clarifies the developmental trend of the literature from subjective judging toward technology-assisted and data-driven assessment. The findings are expected to inform future efforts to build standardized, interpretable, and comparable assessment frameworks that can better integrate technical measurement with aesthetic evaluation in DanceSport.

## 2 Method

### 2.1 Study design: Human-In-The-Loop framework

This study was designed as a systematic review enhanced by Human-In-The-Loop topic modeling. Human-In-The-Loop (HITL) review can combine the transparency of systematic review with the efficiency of machine-assisted text mining, especially when researchers need to identify relevant patterns across a dispersed interdisciplinary literature (Bhutoria, 2022). In this framework, the systematic review procedure established the eligible evidence base through PRISMA-informed literature search, eligibility assessment, screening, and data extraction. LDA topic modeling was then used as an exploratory computational aid for thematic mapping rather than as a confirmatory or standalone source of evidence. Specifically, LDA topic modeling supported the identification of broad thematic structures and stage-based thematic transitions, thereby addressing the review questions concerning the main research themes and their developmental patterns. Qualitative content analysis of the full texts then provided the main interpretive procedure for examining concrete assessment practices, including assessment methods, assessment targets, assessment technologies, data sources, validation designs, and application contexts. In this design, machine-assisted modeling and researcher-led content analysis were not treated as separate procedures, but as complementary components of an integrated HITL framework: LDA provided macro-level thematic cues, while qualitative coding contextualized, checked, and extended these cues through domain-specific interpretation and methodological synthesis.

This design is methodologically appropriate for the present study for three reasons. First, DanceSport assessment is an interdisciplinary topic involving sport science, dance studies, psychology, biomechanics, computer vision, motion analysis, and technology-supported evaluation. Such a dispersed evidence base can be difficult to organize through manual reading alone. LDA topic modeling is a widely used unsupervised method for identifying latent thematic structures in text corpora (Blei et al., 2003; Jelodar et al., 2019), and previous research has shown that machine-assisted text mining can reduce the workload involved in the screening and organization stages of systematic reviews (Miwa et al., 2014). Second, machine-generated topics alone do not provide sufficient conceptual interpretation, because probabilistic word clusters do not automatically correspond to analytically meaningful categories (Nikolenko et al., 2017). Human interpretation therefore remains essential for validating topic meaning, refining category boundaries, and linking statistical patterns to the substantive questions of DanceSport assessment research. Third, qualitative content analysis can identify implicit methodological information that topic modeling cannot reliably capture, such as validation logic, assessment procedures, data-source characteristics, and the practical application of assessment systems. This logic is consistent with recent evidence-synthesis discussions suggesting that automated tools are most appropriate for supporting mechanical aspects of review work, while complex interpretive judgments require human oversight (Gong et al., 2026). In this way, the HITL framework combined computational scalability with interpretive rigor, allowing the review to integrate quantitative thematic mapping with detailed qualitativesynthesis.

### 2.2 Procedure

#### 2.2.1 Literature search and selection

##### 2.2.1.1 Literature search

The literature search followed the Preferred Reporting Items for Systematic Reviews and Meta-Analyses (PRISMA 2020) guidelines to enhance transparency and reproducibility (Page et al., 2021). The search was conducted on the Web of Science Core Collection on August 1, 2024. The following citation indexes were searched: Science Citation Index Expanded, Social Sciences Citation Index, Arts and Humanities Citation Index, and Emerging Sources Citation Index. The publication time span was limited to 2000–2024. Only English-language journal articles that had reached the final publication stage were included. Conference abstracts, proceedings papers, reviews, book chapters, and non-peer-reviewed publications were excluded.

The Web of Science Core Collection was selected because it provides curated journal records across the sciences, social sciences, arts, and humanities, and has been widely used in bibliometric and systematic reviews to capture internationally visible peer-reviewed research activity (Kee et al., 2019). This database was considered appropriate for the interdisciplinary scope of DanceSport assessment, which spans sport science, dance studies, psychology, movement analysis, and technology-assisted evaluation.

The search strategy combined DanceSport-related terms with assessment-related terms. Searches were performed in the Web of Science Topic field using the Boolean structure: TS = (dance-related keyword) AND TS = (assessment OR performance). Dance-related keywords included “sport dance,” “DanceSport,” “competitive ballroom dancing,” the five International Standard dances, and the five International Latin-American dances (the complete search strings and outputs are provided in Supplementary Table S2). The classification of DanceSport categories and dance styles followed definitions commonly adopted in recent DanceSport research (Dai et al., 2024; Liu et al., 2024). The database, citation indexes, search date, time span, language restrictions, document-type restrictions, and screening parameters are reported in Supplementary Table S1.

**Figure 1:**
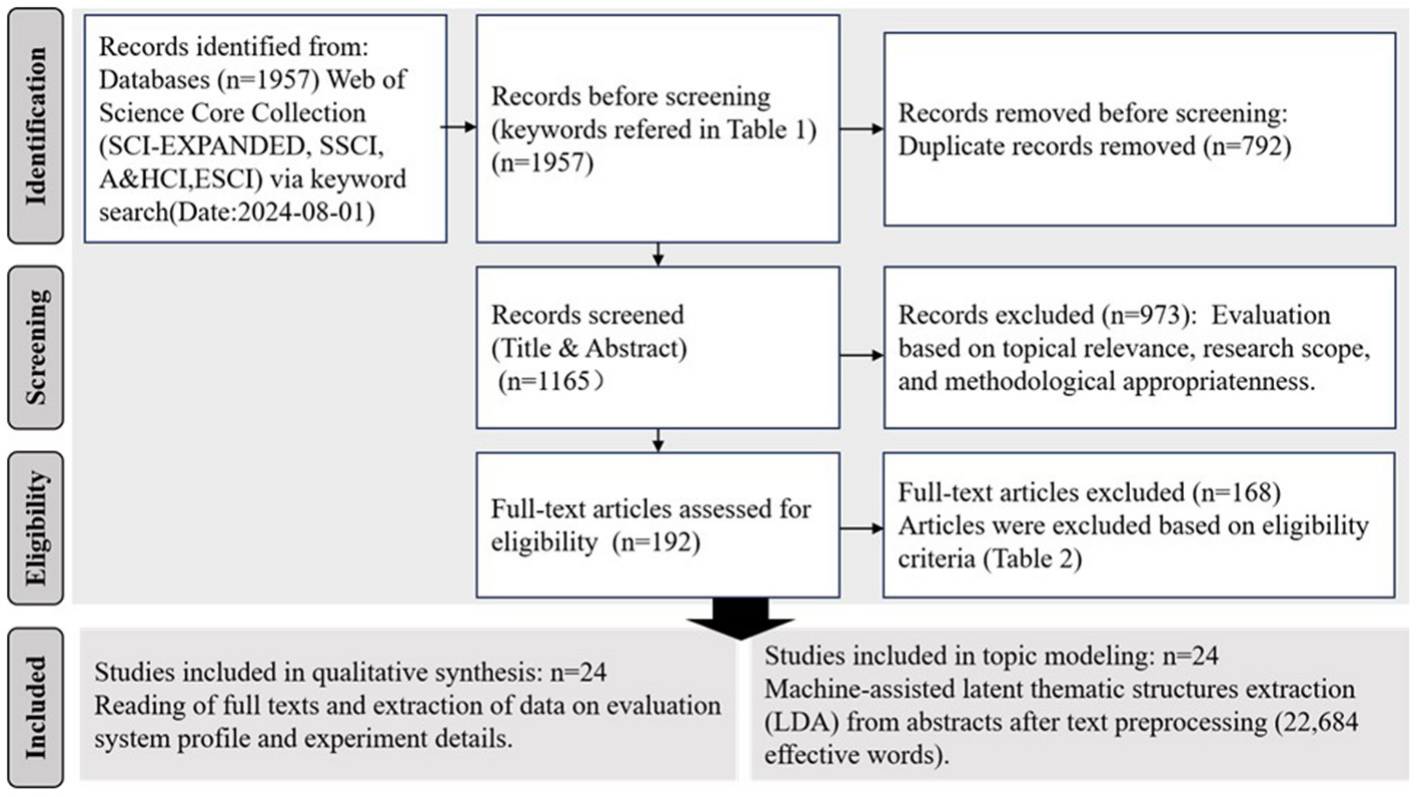
PRISMA flow diagram of the study selection process.

All records retrieved from the 26 searches were exported from Web of Science and imported into reference-management software for duplicate checking and citation management. The deduplicated records were then organized in a spreadsheet for title-and-abstract screening, eligibility assessment, and data extraction. The number of records identified, screened, excluded, assessed at full text, and finally included is reported in the PRISMA flow diagram in Section 3 (Figure 1).

##### 2.2.1.2 Eligibility criteria

The eligibility criteria were informed by an adapted Population/Problem, Intervention, Comparison, Outcomes, and Study Design (PICOS) framework and previous review studies on dance assessment and performance evaluation methods (Quadrado et al., 2022). In this review, PICOS was used as a structured guide to clarify the population or problem, assessment approach, comparison logic, outcome relevance, and study context of potentially eligible studies, rather than as a clinical-intervention framework (Daly et al., 2022).

Given the interdisciplinary scope of DanceSport assessment research, the review distinguished between two types of eligible studies. First, DanceSport-specific studies were included when they explicitly examined DanceSport, competitive ballroom dancing, International Standard Dance, International Latin Dance, or one of the DanceSport styles included in the search strategy. Second, studies not explicitly labeled as DanceSport were retained only when their assessment logic, movement-analysis procedure, or validation design was directly transferable to DanceSport performance assessment. Such studies were required to address performance-related evaluation, movement quality, posture or motion analysis, expert scoring, technology-supported assessment, or validation procedures in dance or comparable aesthetic-motor performance contexts. Studies using technical measurement tools without a clear connection to performance evaluation were excluded. The final eligibility criteria are summarized in Table 2.

**Table 2:** Inclusion and exclusion criteria informed by an adapted PICOS framework.

PICOS element	Inclusion criteria	Exclusion criteria
Population/problem	DanceSport performers, judges, coaches, or comparable aesthetic-motor performance contexts with direct relevance to DanceSport assessment.	Clinical, rehabilitation, elderly, general fitness, or non-performance-related populations without assessment relevance.
Intervention/assessment approach	Judge-based, expert-rated, software-assisted, motion-analysis, image-based, sensor-based, or technology-supported assessment approaches.	Isolated measurement techniques without evaluative purpose or performance-assessment logic.
Comparator	Comparisons across groups, performance levels, assessment methods, expert scores, algorithmic outputs, or validation conditions, where applicable.	Studies without evaluative, comparative, validation, or classification components.
Outcomes	Assessment validity, reliability, scoring accuracy, movement quality, posture or motion features, system performance, evaluation indicators, or assessment quality.	Studies not addressing performance quality, judging logic, movement assessment, or evaluation outcomes.
Study design/context	Empirical peer-reviewed journal articles in recreational, educational, laboratory, training, or competitive performance contexts.	Conference abstracts, proceedings, reviews, book chapters, non-peer-reviewed publications, purely theoretical papers, or non-transferable studies.

##### 2.2.1.3 Study selection and screening reliability

The study selection process followed the PRISMA 2020 procedure and consisted of duplicate removal, title-and-abstract screening, and full-text eligibility assessment. All records retrieved from Web of Science were exported in BibTeX format and imported into Zotero (Corporation for Digital Scholarship, 2024) for duplicate checking, citation management, and record organization. The remaining records were collated in Microsoft Excel (Microsoft Corporation, Redmond, WA, United States) using a structured screening and extraction form.

Two reviewers independently screened the titles and abstracts against the predefined eligibility criteria. Records that were clearly outside the review scope were excluded, whereas records that were potentially relevant, ambiguous, or methodologically transferable to DanceSport assessment were retained for full-text assessment. The full texts of potentially eligible articles were then assessed by the same two reviewers. Particular attention was given to whether each study was DanceSport-specific or methodologically transferable according to the transferability rule described above.

Disagreements were resolved through consensus discussion. When an immediate agreement could not be reached, the third author was involved in the discussion, and the final decision was made by returning to the PICOS-informed eligibility criteria and the study's assessment aim, methodological procedure, and relevance to DanceSport performance evaluation. Formal inter-rater agreement statistics were not calculated; instead, all uncertain or borderline cases were discussed until consensus was reached. The final screening decisions were recorded and used to construct the PRISMA flow diagram (Figure 1).

After study selection was completed, bibliographic and methodological information was extracted using the structured extraction form. The abstracts of the final included studies were used for exploratory LDA topic modeling, whereas the full texts were used for qualitative content analysis and methodological quality appraisal.

#### 2.2.2 Exploratory LDA topic modeling and thematic mapping

##### 2.2.2.1 Text preprocessing

Standardized text preprocessing was conducted on the title and abstracts of the selected studies prior to topic modeling. The texts were cleaned by removing non-English items, numerical tokens, punctuation, and publisher- or copyright-related content, and were subsequently tokenized and normalized using the Natural Language Toolkit (NLTK, version 3.5). To improve topic interpretability and reduce noise, standard English stopwords were combined with a customized stopword list, domain-specific terminology was preserved through a customized vocabulary, and synonymous expressions were normalized into unified forms. Part-of-speech filtering was also applied to retain substantively meaningful terms relevant to the review focus. Following established text-mining procedures, term frequency–inverse document frequency (TF-IDF) was calculated to support the identification of salient lexical items and facilitate corpus interpretation (Ravikumar et al., 2015). The detailed preprocessing rules, lexical normalization procedures, and corpus summary are reported in Supplementary Table S3.

##### 2.2.2.2 LDA topic modeling and topic selection

Based on the preprocessing corpus, a bag-of-words representation was constructed and used as the input for the LDA model. Following the approach of Chen et al. (2024), we utilized the Gensim library in Python to build the topic model. The optimal number of topics was determined by jointly considering perplexity, coherence, and researcher interpretability (Zhao et al., 2015). Perplexity and coherence were used as diagnostic indicators rather than definitive criteria, because topic modeling in a strictly screened review corpus may be sensitive to document number, preprocessing decisions, and topic-number selection. Candidate topic numbers from 1 to 20 were tested. Topic perplexity assesses the quality of probability distribution or prediction samples (see Supplementary Formula S1), with lower perplexity values indicating a more stable model structure and clearer topic separation (Arun et al., 2010). Coherence, on the other hand, measures the distribution distance between topics (Stevens et al., 2012; see Supplementary Formula S2). We employed the coefficient of variation (C_V) method with a sliding window to calculate coherence. Higher coherence values indicate better topic distinction and improved clustering performance (Chen et al., 2024). We selected integers in the range of 1–20 as potential topic numbers and computed their respective perplexity and coherence scores. The final number of topics was determined by visually comparing the perplexity and coherence curves and identifying an appropriate balance between model stability and interpretability (the diagnostic curves are shown in Supplementary Figure S1).

The LDA models were fitted using 20 passes, a fixed random seed of 42, and the hyperparameters α and β were set to 0.1 and 0.01, respectively. Other technical parameters not manually specified were retained at the default Gensim settings. In addition, Topic intensity (T_*k*_) was calculated as the cumulative document-topic probability of each topic and was interpreted as a descriptive indicator of relative prominence within the included corpus (see Supplementary Formula S3). The full model settings are provided in Supplementary Table S4.

Using the predefined number of topics, we will implement LDA topic analysis and generate the topic distributions. To address this, the research team examined the topic-word lists and document-topic distributions, discussed the thematic meaning of each topic, and assigned final topic labels according to the characteristics of DanceSport assessment research. Consensus was reached among all co-authors through iterative discussion. To reduce interpretive bias, two external researchers with no conflict of interest and no prior involvement in the project were also invited to independently evaluate the topic classification results.

An overall LDA model was first constructed for the full corpus. To support longitudinal thematic analysis, additional LDA models were estimated for publication subsets derived from publication trends over time. The same modeling procedure was applied across the overall corpus and the temporally grouped subsets to ensure methodological consistency.

##### 2.2.2.3 Similarity-based thematic transition analysis

Based on the temporal-stage LDA models, similarity-based thematic transition analysis was conducted to describe lexical and conceptual continuity between topics from adjacent stages. Topics that emerge at similar developmental stages often exhibit substantial lexical overlap and conceptual continuity. To quantify these relationships, cosine similarity was used to measure the lexical proximity between topic vectors (Qian et al., 2020). Based on existing studies, we use the average cosine similarity of adjacent temporal stages as the threshold (Refer to the Supplementary Formula S4). The visualization threshold was set at cosine similarity ≥0.1, corresponding to at least one shared top keyword under the binary top-keyword representation. Topic pairs with a similarity value of 0 were excluded from the Sankey visualization. Link thickness was proportional to the cosine similarity value, with higher values indicating stronger lexical-conceptual proximity between adjacent-stage topics. Only topic pairs with cosine similarity values above the predefined threshold were visualized in the Sankey diagram. The thickness of each Sankey link is proportional to the cosine similarity between the source topic and the target topic. This approach allowed the study to trace thematic transition and transformation across the development of DanceSports assessment research (Jung and Segev, 2022). These transitions were interpreted as indicative thematic continuities rather than deterministic evolutionary relationships.

##### 2.2.2.4 Trustworthiness and interpretive validation of topic modeling

To strengthen the interpretive credibility of the exploratory topic modeling results, we adapted a trustworthiness strategies used in qualitative thematic analysis, including researcher triangulation, peer debriefing, audit-trail documentation, and returning to the raw data to assess referential adequacy (Nowell et al., 2017). First, preprocessing decisions, synonym-normalization rules, model parameters, topic-word lists, document-topic distributions, topic-intensity values, and similarity matrices were archived to document the analytical process. Second, the research team reviewed the topic-word lists and representative documents associated with each topic, discussed the conceptual meaning and boundaries of each topic, and assigned topic labels through consensus. Third, topic labels and thematic transitions were checked against the original abstracts and, where necessary, the full texts of the included studies. Finally, the topic interpretations were compared with the qualitative content analysis results to determine whether the computationally generated topics corresponded to concrete assessment modes, technologies, validation designs, or methodological limitations. Accordingly, LDA results were treated as indicative thematic structures and were not used as standalone evidence unless supported by full-text coding and methodological synthesis.

#### 2.2.3 Qualitative content analysis

A structured coding framework was developed deductively from the research questions, the PICOS-informed eligibility criteria, and the variables required for the content-analysis tables. The framework was then refined inductively during full-text reading when additional recurring features emerged. We extracted information on two dimensions: the basic profile of the assessment system (category of dance, system name, assessment mode, object of judgment, evaluation category, and assessment technology) and the experimental designs used to validate assessment systems (participants, assessment data source, dance movement, and comparison between groups).

To improve coding consistency, an initial coding form with operational definitions was developed before full-text coding. Two authors independently coded the included studies using the structured form. Coding results were then compared item by item. Disagreements were resolved through discussion, and when consensus could not be reached immediately, the third author participated in the adjudication. Ambiguous classifications were resolved by returning to the study's stated assessment aim, data source, assessment procedure, and validation logic. Because a single study could involve more than one assessment method, target, technology, data source, or validation strategy, multiple codes were allowed where appropriate; therefore, category counts were not treated as mutually exclusive.

The final coding results were collated in a spreadsheet and used to construct the content-analysis tables reported in Section 3. The coding framework, operational definitions, and coding examples are provided in Supplementary Table S6.

#### 2.2.4 Methodological quality appraisal

Finally, a simplified methodological quality appraisal was conducted to evaluate the reporting transparency and methodological comparability of the included studies. This appraisal was not used to exclude studies, but to inform the interpretation of methodological transparency, validation heterogeneity, and evidence comparability. The appraisal included six criteria: sample characteristics, validation design, comparison group or reference standard, data source clarity, indicator definition, and method reproducibility. Each criterion was rated as “Yes,” “Partial,” or “No.” For descriptive summarization, Yes, Partial, and No were assigned scores of 2, 1, and 0, respectively, resulting in a maximum score of 12. Studies scoring 10–12 were classified as having high reporting quality, those scoring 6–9 as moderate reporting quality, and those scoring 0–5 as low reporting quality. The full appraisal items are provided in Supplementary Table S7.

## 3 Results

### 3.1 Study selection, publication trends, and methodological characteristics

The study selection process is illustrated in Figure 1 and follows the procedure described in Section 2.2.1. A total of 1,957 records were identified from the Web of Science Core Collection. After duplicate removal, 1,165 records were screened by title and abstract, of which 973 were excluded because they did not meet the topical, methodological, or scope-related criteria. The remaining 192 full-text articles were assessed for eligibility, and 168 were excluded according to the predefined eligibility criteria. Finally, 24 studies were included in the review. All these included studies were used for qualitative content analysis based on full-text reading, and their abstracts were used for exploratory LDA topic modeling after text preprocessing.

**Figure 2:**
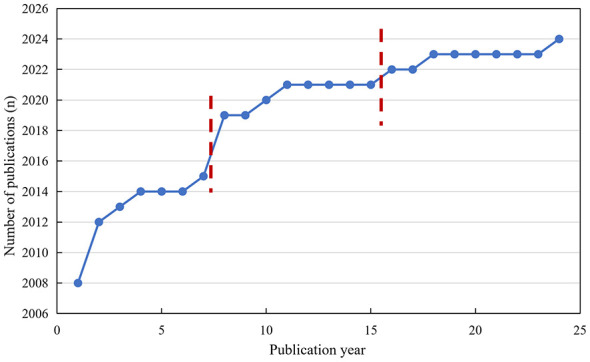
Publication trends in DanceSport assessment research.

The publication trend is presented in Figure 2. DanceSport assessment research remained sparse during the early part of the review period but showed a gradual increase after 2008, indicating growing scholarly interest in performance evaluation. Based on the publication timeline and volume, the development of this field can be divided into three stages: the exploration stage (2008–2014), the growth stage (2014–2020), and the expansion stage (2020–2024).

Among the 24 included studies, 14 were directly DanceSport-specific, four focused on particular dance styles, and six were methodologically transferable studies. This distribution indicates that the current evidence base is not limited to studies explicitly labeled as DanceSport, but also includes research whose assessment logic, movement-analysis procedures, or validation designs are relevant to DanceSport performance evaluation. The transferable studies were therefore retained to capture methodological developments across adjacent aesthetic-motor performance contexts, but they were interpreted cautiously because their relevance to DanceSport was indirect.

The simplified methodological quality appraisal showed that 17 studies were rated as high reporting quality, three as moderate reporting quality, and four as low reporting quality, with an average score of 9.875 out of 12. The study-level appraisal results are provided in Supplementary Data S1. Overall, most studies clearly reported data sources, assessment aims, and the technical or evaluative procedures used in the study. However, reporting was less consistent for validation design, comparison groups or reference standards, explicit definitions of assessment indicators, and reproducibility of computational or sensor-based procedures. These results indicate that the reviewed literature provides useful methodological evidence for understanding DanceSport assessment practices, but the comparability of findings remains constrained by uneven reporting standards and heterogeneous validation designs. Therefore, the methodological quality appraisal was used to contextualize the synthesis rather than to exclude studies from the review.

### 3.2 Exploratory topic modeling results

#### 3.2.1 Major themes in DanceSport assessment research

After preprocessing, the LDA corpus consisted of the abstracts of the 24 included studies, with 22,684 characters before preprocessing, 20,242 words after preprocessing, and 1,235 effective lexical items identified through word-frequency analysis. As shown in Supplementary Figure S1, the model with four topics showed relatively low perplexity and high coherence, with clear inflection points in both curves. Therefore, *K* = 4 was selected as the final topic number for the exploratory LDA model.

The LDA results identified four major thematic clusters: 3D dance motion perception (T1), Dance posture and motion evaluation (T2), image analysis of dynamic dance performance (T3), and validation of dance evaluation (T4). Table 3 presents the top 10 keywords and topic intensity values for each topic.

**Table 3:**
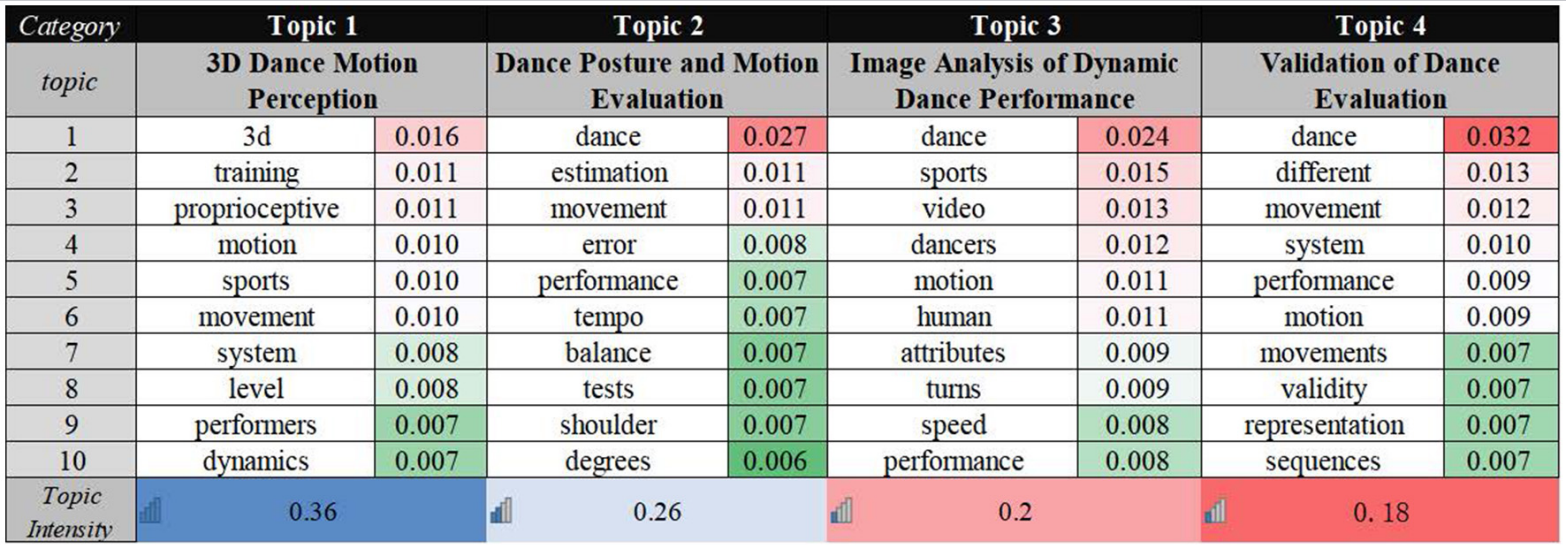
Probability distribution and intensity of topics and keywords.

T1 (T_*k*_ = 0.36) and T2 (T_*k*_ = 0.26) exceeded the mean probability (Mean T_*k*_ = 0.25), suggesting that the most visible strand of DanceSport assessment research is concentrated on movement description, posture analysis, and technical differentiation. The two topics differ in their emphasis. T1, characterized by keywords such as “3d,” “training,” “proprioceptive,” “movement,” and “system,” reflects a research focus on perceiving and modeling dance movement through spatial, proprioceptive, and motion-related information, emphasizing motion perception and representation. In contrast, T2, represented by keywords including “movement,” “error,” “tempo,” “balance,” “tests,” and “shoulder,” is more directly concerned with evaluating the accuracy and quality of bodily movement. T3 showed a relatively lower intensity (T_*k*_ = 0.20) and captures studies that use visual or video-based data to analyze observable features of dance performance, particularly dynamic motion, speed, turns, and human movement attributes. Its keyword structure indicates that image-based analysis has become an important approach for translating visible performance characteristics into measurable data.

T4 (T_*k*_ = 0.18) captures studies concerned with evaluation validity, representation, and the organization of assessment sequences. Representative keywords include “validity,” “representation,” and “sequences.” Although its topic intensity was lower than that of technology-driven assessment approaches, its presence indicates that validation and evaluation logic remain a distinct concern within the field.

#### 3.2.2 Similarity-based thematic transition analysis

Stage-specific LDA models were further estimated to examine how thematic emphases changed across the three publication stages. As shown in Supplementary Figure S1, the optimal number of topics was determined as *K* = 2 for the exploration stage (Stage 1), *K* = 3 for the growth stage (Stage 2), and *K* = 5 for the expansion stage (Stage 3). Table 4 reports the topic labels, topic intensity values, and top keywords for each stage.

**Table 4:** Stage-specific LDA topics, topic intensity values, and top keywords.

Period	Number	Topic	Topic intensity	Keywords
Stage 1	1	Sports training and testing systems	0.50	sports; training; dance; proprioceptive; balance; tests; competition; system; test; level
	2	Scoring standard and evaluation validity	0.50	judges; judging; agreement; validity; marks; criteria; scale; motion perfection
Stage 2	1	Sports and dance posture datasets and image analysis	0.25	datasets; dance; sports; postures; image; yoga; Syd-net; CNNs; performance; accuracy
	2	Dance motion performance and movement model analysis	0.50	turns; performers; lower; reverse; couples; dancers; movement; speed; model; performance
	3	Motion capture and dance system performance evaluation	0.25	dance; mocap; performance; kinematic; system; RMSE; reactive; validity; analysis; systems
Stage 3	1	Motor skills and anticipatory performance	0.11	expertise; motor; semantic; activity; performance; temporal; actions; responses; level; anticipatory
	2	Motion errors and angle estimation methods	0.33	error; motion; shoulder; estimation; angle; movement; degrees; performance; rotation; methods
	3	Movement sequences and aesthetic analysis	0.22	movement; dance; movements; sequences; features; different; velocity; aesthetics; system; karate
	4	Dance dynamics and precision evaluation	0.22	dynamics; dance; judgments; gait; attributes; physical; learning; precision; technique; perceivers
	5	Dance style and musical rhythm information processing	0.11	dance; ballroom; information; style; tempo; large; music; rhythmic; meter; audio

In the exploration stage, the topic structure was evenly divided between Sports Training and Testing Systems (T_*k*_ = 0.5) and Scoring Standard and Evaluation Validity (T_*k*_ = 0.5). The first topic, represented by keywords such as “training” and “balance,” reflects an early focus on training-related assessment and basic performance testing. The second topic, characterized by terms such as “judging” and “validity,” highlights concerns with scoring standards, judging agreement, and evaluation validity.

In the growth stage, the topic structure became more differentiated. Dance motion performance and movement model analysis showed the highest topic intensity (T_*k*_ = 0.5), indicating increased attention to movement modeling and dynamic performance characteristics. The other two topics, sports and dance posture datasets and image analysis (T_*k*_ = 0.25) and motion capture and dance system performance evaluation (T_*k*_ = 0.25), suggest the emergence of posture datasets, image-based analysis, motion capture, and system-level evaluation as distinct methodological directions.

In the expansion stage, the topic structure became more diversified. motion errors and angle estimation methods had the highest topic intensity (T_*k*_ = 0.33), reflecting growing attention to technical error detection and angle-based movement estimation. Movement sequences and aesthetic analysis (T_*k*_ = 0.22) and dance dynamics and precision evaluation (T_*k*_ = 0.22) indicate a shift toward movement sequences, dynamic attributes, precision, and aesthetic features. The remaining topics, motor skills and anticipatory performance (T_*k*_ = 0.11) and dance style and musical rhythm information processing (T_*k*_ = 0.11), represent additional but less prominent interests in motor expertise, anticipatory responses, and rhythm-related information processing.

**Figure 3:**
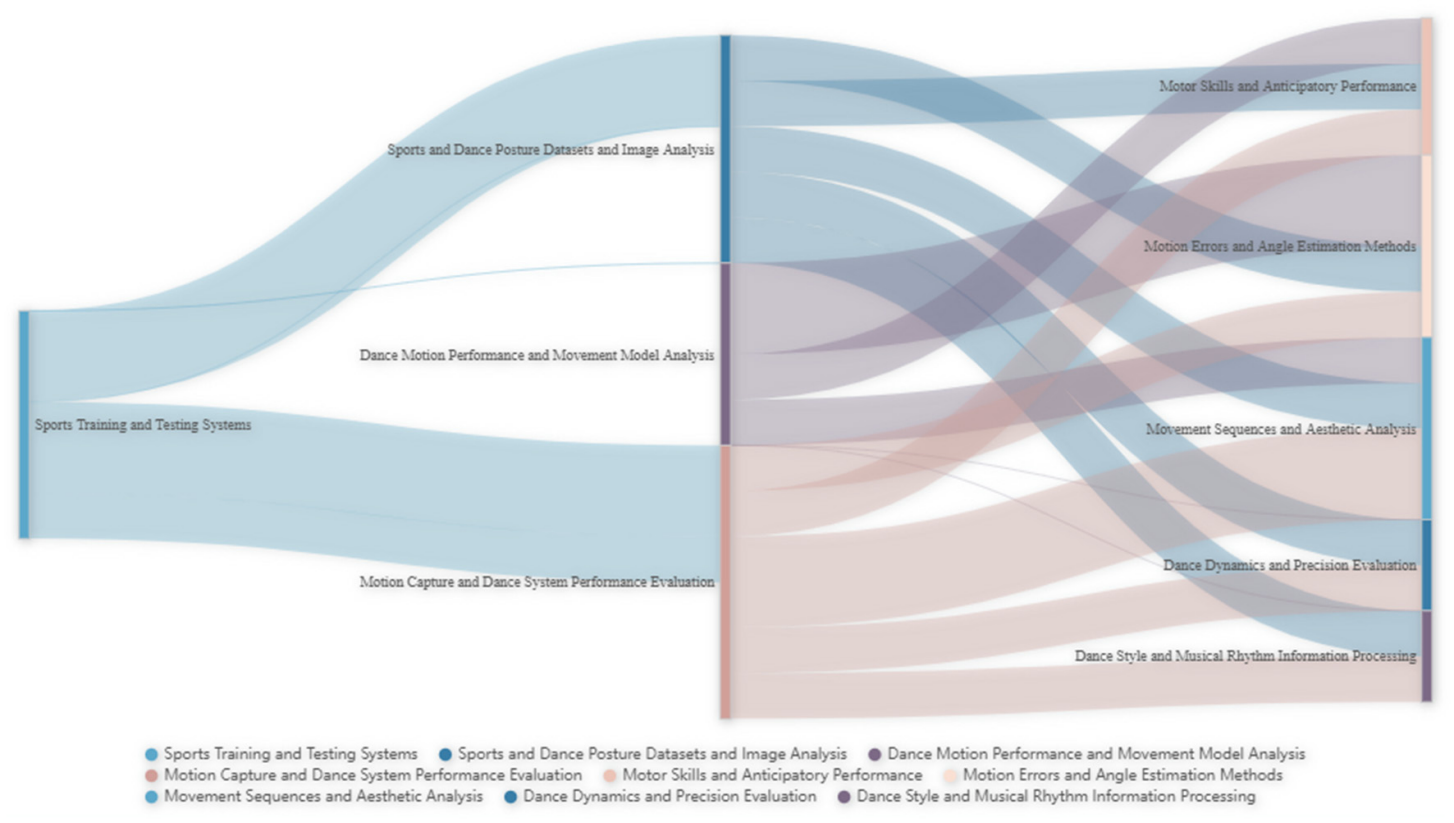
Similarity-based thematic transitions across developmental stages.

Figure 3 presents a Sankey diagram illustrating the associations among the stage-specific topics reported in Table 4. In this diagram, the horizontal axis represents the three developmental stages, and the nodes represent the stage-specific LDA topics. The links indicate lexical-conceptual similarity between adjacent-stage topics. Link thickness corresponds to the cosine similarity value, with thicker links indicating stronger similarity between topics. The complete similarity matrix and retained Sankey links are provided in Supplementary Table S5.

The Sankey diagram shows that early topics related to training, testing, scoring standards, and judging validity were subsequently connected to topics emphasizing movement modeling, posture datasets, image analysis, and motion-capture-based system evaluation. In the most recent stage, these themes further diversified into motion-error estimation, angle-based analysis, movement sequences, dance dynamics, precision evaluation, and rhythm-related information processing. This transition pattern suggests that DanceSport assessment research has gradually moved from general concerns with judging validity and performance testing toward more specific technology-assisted approaches to movement analysis and system-supported evaluation.

### 3.3 Qualitative content analysis results

#### 3.3.1 Assessment system profiles

To address RQ3, this section summarizes the assessment system profiles of the included studies. Table 5 provides the study-level coding results, while the text below synthesizes the major patterns across dance categories, assessment methods, targets, and technologies.

**Table 5:** Basic profiles of DanceSport assessment systems.

References	Category of dance	Assessment method	Assessment targets	Assessment technology
Amoruso et al. (2014)	Tango	Judge-based evaluation	Assessing the expertise of athletes/referees	Event-Related Potentials (ERPs) and self-assessment questionnaires for assessing expertise level
Anderlucci et al. (2021)	DanceSport (Latin)	Judge-based evaluation	Optimizing judges' scoring patterns	Many-facet Rasch measurement framework
Beck et al. (2024)	DanceSport	Software-assisted evaluation	Planning and assessing dance choreographies	Sports visualization implemented as a web-based prototype using React and D3.js with responsive design
Bera et al. (2023)	Samba	Software-assisted evaluation	Postures recognition	Deep convolutional neural networks (CNNs)
Chavoshi et al. (2014)	Samba	Software-assisted evaluation	Regular and cyclic movements of the Samba dance	Location-aware technologies, map algebra with relative motion matrices, and dynamic time warping applied to time series of motion attributes
Clarke et al. (2021)	Contemporary, jazz	software–hardware integrated systems	Static and dynamic field balance tests	Star excursion balance test, modified Romberg test, airplane test, BioSway balance system (Biodex, USA), and dance-specific pirouette test
Fang and Sun (2021)	DanceSport	software–hardware integrated systems	Action type recognition	Human motion recognition technology, including software and hardware components: Alibaba Cloud Elastic Compute Service (ECS), CPU, hard disk, infrared recognizer, and signal receiver
Gallo et al. (2022)	Not specified	Software-assisted evaluation	Visualization, analysis, annotation, and record of motor practice, optimization of reference information for decision-making in DanceSport practice	Two software components: a tool to extract frames from a reference or focal video (FindFrame) and a tool to bundle images for presentation, assessment, and annotation (preparePDFs)
Geukes et al. (2023)	Modern, contemporary, or jazz dance	Judge-based evaluation	Dancers' physical appearance, technique, and expression; optimization of reference information for decision making	A naturalistic lens model (consist of a developed items scale)
Han et al. (2015)	DanceSport	Judge-based evaluation	Multi-joint proprioceptive ability	Active movement extent discrimination apparatus (AMEDA)
Kulis and Gajewski (2022)	DanceSport (Vinnese Walz)	Judge-based evaluation	Body movement recognition; optimization of reference information for decision-making	Kinematic evaluation using four three-component accelerometers with a digital recorder to measure angular velocities of the thoracic spine and pelvic girdle sections
Niewiadomski et al. (2019)	Sports (karate)	software–hardware integrated systems	Movement quality in full-body physical activities and biomechanical efficiency; quantitative reference for judges' decision-making	Automated approximate measure based on biomechanical analysis, shape, and intrapersonal synchronization
Ofori et al. (2021)	Dance training (specific dance type not reported)	software–hardware integrated systems	Balance kinematics and mobility during treadmill-based perturbation and dance training	Inertial-sensor kinematic data, including an eight-camera motion-capture system with 29 reflective markers and seven inertial sensors
Oh et al. (2014)	Samba	Software–hardware integrated systems	Real-time motion capture of the samba for real-time evaluation	Motion capture based on data-driven 3D human pose reconstruction, wireless camera sensor networks, human-motion constraints, human joint models, local pose space methods, and pseudo-pose generation
Cui and Guo (2023)	DanceSport	Software-assisted evaluation	Video analysis and intelligent signal processing, diagnosis, and scoring of DanceSport training items	Complex background modeling algorithm based on region segmentation and real-time moving-target tracking algorithm based on dynamic model switching
Oliveira et al. (2012)	Popular dance styles (samba, American Charleston style dance)	Software-assisted evaluation	Structure of dance gestures according to musical meter; motion-capture system for dance style recognition	Motion-capture system for data acquisition and topological gesture analysis (TGA) for synthesizing dance sequences
Orlandi et al. (2020)	Dance (choreography duet)	Software-assisted evaluation	Dance aesthetics, including motion energy, acceleration, velocity per limb, smoothness, and entropy	Assessment movement kinematics complexity and linear mixed-effects modeling
Prosen et al. (2013)	Viennese Waltz	Software–hardware integrated systems	Motion and timing of turns	Time-motion analysis
Schuller et al. (2008)	DanceSport	Software-assisted evaluation	Ballroom-dance style recognition, meter recognition, and tempo detection.	Data-driven rhythm analysis approach
Stancin and Tomazic (2021)	DanceSport (jazz)	Software–hardware integrated systems	Dance tempo	Single Leg-attached 3D accelerometer: MbientLab MetaMotionR wearable device
Sun and Wu (2023)	DanceSport	Software–hardware integrated systems	Categorization of DanceSport performers' emotions and recognition of emotions in dance videos	Arousal–valence emotion model, fusion neural network model (FUSNN), and DanceSport movement recognition using Kinect 3D sensor
Yang and Hsieh (2023)	Tango	Software-assisted evaluation	Categorization of DanceSport performers' emotions and recognition of emotions in dance videos	Mimicking personal gait dynamics using 12-dimensional time series derived from four accelerometer sensors in the MAREA database
Zhu et al. (2023)	Samba	Hardware-assisted evaluation	Accurate shoulder-angle joint estimation	Inertial measurement units (IMUs)-based shoulder-angle estimation and analytical model based on quaternions and rotation vectors to decouple and quantify two key error factors
Premelč et al. (2019)	DanceSport	Judge-based evaluation	Reliability and validity of the new judging system in DanceSport, including technical and artistic components	New judging system consists of technical and artistic parts across 10 level

##### 3.3.1.1 Dance categories covered

The included studies showed uneven coverage across DanceSport categories. Seven studies addressed DanceSport as a general category without distinguishing between International Standard Dance and International Latin Dance. Four studies focused on International Standard Dance styles, including Tango and Viennese Waltz, while five studies focused on International Latin Dance styles, mainly Samba and Latin Dance more broadly. One additional study examined Samba together with American Charleston and was therefore treated as a Latin-related mixed dance-style study rather than a purely DanceSport-specific case (Oliveira et al., 2012). No included study directly compared International Standard Dance and International Latin Dance within the same validation design or examined whether assessment indicators, posture-analysis priorities, movement-recognition methods, or technical tools performed differently across the two categories.

##### 3.3.1.2 Assessment targets and methods

DanceSport assessment methods were classified into three mutually exclusive modes (judge-based evaluation, software-assisted evaluation, software–hardware integrated systems), with one additional hardware-assisted study discussed separately.

The first category comprised studies that adopted judge-based evaluation as their primary assessment mode (*N* = 6). These studies primarily addressed assessment targets related to judging quality and scoring reliability, including the evaluation of judges' expertise (Amoruso et al., 2014), the optimization of scoring models (Anderlucci et al., 2021), and the provision of structured reference information for decision-making (Geukes et al., 2023; Han et al., 2015; Kulis and Gajewski, 2022). In addition, some studies examined the reliability and validity of existing judging systems (Premelč et al., 2019). Judge-based studies were primarily concerned with improving or explaining expert adjudication rather than replacing it.

Second, studies developed software-based approaches to DanceSport assessment (*N*=9). These studies primarily targeted movement recognition and posture analysis, as well as biomechanical efficiency and technical execution. For example, several studies focused on analyzing biomechanical and motion-related data (Prosen et al., 2013; Stancin and Tomazic, 2021; Sun and Wu, 2023). Other studies extended software applications to additional assessment targets, including aesthetic analysis (Orlandi et al., 2020), dance style recognition (Schuller et al., 2008), and gait-based movement recognition in Tango (Yang and Hsieh, 2023). These studies indicate that software-assisted assessment has mainly developed around the conversion of visible or recorded performance features into analyzable data.

Third, eight studies integrated hardware devices with data processing systems to support DanceSport assessment. These systems typically relied on physiological signal acquisition, motion capture, or sensor-based data collection, including wearable sensors (Ofori et al., 2021; Stancin and Tomazic, 2021; Sun and Wu, 2023), infrared motion detectors (Fang and Sun, 2021), and time-motion or 3D posture analysis technologies (Oh et al., 2014; Prosen et al., 2013). Within this mode, assessment targets were mainly related to biomechanical efficiency, technical execution, and movement analysis. One additional study was classified as hardware-assisted evaluation because it focused specifically on IMU-based shoulder-joint angle estimation rather than a broader software–hardware assessment system (Zhu et al., 2023). Overall, most studies used hardware primarily for data acquisition in combination with software-based analysis (Clarke et al., 2021; Niewiadomski et al., 2019).

##### 3.3.1.3 Assessment technologies

The assessment methods described above were implemented through five main types of technologies: visual and video-based analysis, motion capture and kinematic measurement, wearable or sensor-based data acquisition, computational recognition and modeling, and expert scoring or psychometric modeling. These technology categories were not mutually exclusive, because several studies combined multiple tools within the same assessment system.

Several studies used visual data to examine DanceSport performance, including movement analysis, posture detection, and video-based scoring. These approaches included sports photography and time-motion analysis (Prosen et al., 2013), data-driven 3D posture analysis (Oh et al., 2014), infrared-based image recognition (Fang and Sun, 2021) and video assessment systems for DanceSport training and scoring (Cui and Guo, 2023). Visual approaches were also used in studies of dance aesthetics and style-related features (Orlandi et al., 2020; Schuller et al., 2008). These studies show that visual technologies were mainly used to transform observable performance features into analyzable movement or posture data.

Another group of studies focused on motion capture and kinematic measurement to quantify movement trajectories, body coordination, and joint-level performance. These studies included analyses of dance gestures and meter-related movement structures (Oliveira et al., 2012), angular velocity and body motion in Viennese Waltz (Kulis and Gajewski, 2022), and movement timing or turn-related analysis (Prosen et al., 2013). Some studies also used these approaches to assess biomechanical efficiency and posture-related performance features (Ofori et al., 2021; Stancin and Tomazic, 2021). This group of technologies was primarily oriented toward measuring technical execution, coordination, balance, timing, and movement accuracy.

Several studies used wearable or sensor-based technologies to collect movement-related data in DanceSport or related performance contexts. These technologies included inertial sensors (Ofori et al., 2021), wearable accelerometers (Stancin and Tomazic, 2021), Kinect-based motion recognition (Sun and Wu, 2023), and IMU-based joint angle estimation (Zhu et al., 2023). In most cases, these devices supported the capture of real-time motion features for subsequent analysis.

A number of studies used computational methods to classify, model, or recognize DanceSport-related movement patterns. These included movement recognition and signal processing models (Fang and Sun, 2021), gait-based recognition in Tango (Yang and Hsieh, 2023), style recognition and rhythm analysis (Schuller et al., 2008), and software systems for posture or performance analysis (Sun and Wu, 2023). Machine learning and algorithm-based approaches also appeared in studies of automated analysis and intelligent scoring support (Cui and Guo, 2023). These methods were mainly applied to recognition, classification, diagnosis, or decision-support tasks rather than to complete adjudication systems.

In addition to data-driven approaches, several studies relied on expert scoring systems or psychometric models. These included the evaluation of dancers' physical appearance, technique, and expression (Geukes et al., 2023), assessments of proprioceptive ability (Han et al., 2015), the analysis of scoring models and judging patterns (Anderlucci et al., 2021), and the evaluation of reliability and validity in judging systems (Premelč et al., 2019). Amoruso et al. (2014) also combined performance evaluation with Event-Related Potential (ERP) and self-assessment measures to examine expertise-related differences in tango performance. These studies show that expert judgment and psychometric modeling remain important for evaluating dimensions that are difficult to capture through movement variables alone.

Taken together, the reviewed technologies show that DanceSport assessment research is most developed in the measurement of visible and quantifiable movement features, including posture, balance, speed, turns, joint angles, rhythm, and movement recognition. In contrast, fewer systems directly operationalized musicality, expressiveness, partner interaction, stylistic interpretation, or the integration of technical scores with formal adjudication logic.

#### 3.3.2 Validation designs of assessment systems

To address RQ4, this section summarizes the studies that reported explicit evaluation procedures (*N* = 21) and focuses on four aspects of their validation designs: data sources, participant types, movement tasks, and inter-group comparisons (see Table 6). Taken together, these studies show substantial variation in how DanceSport assessment systems were tested across research settings.

**Table 6:** Experimental designs used to validate assessment systems.

References	Participants	Validation data sources	Dance movement/task	Comparison between groups
Amoruso et al. (2014)	*N* = 80	Videos of correct and incorrect tango performances (*n* = 330)	Classical steps of tango salon style (ballroom tango): Salida básica, sandwichito, americana, cambio de frente, salida de 40, gancho, sentadita, calesita, barrida and sacada.	Expert Tango dancers, beginner Tango dancers, and naïve participants
Anderlucci et al. (2021)	26 teams and 18 raters	Italian national championship of sportdance (synchro Latin dance)	Technique, choreography, image and show, all measured on a 10 point-scale	Not reported
Beck et al. (2024)	Interview (conducted three times)	Recording of real-time performance	Not reported	Not reported
Bera et al. (2023)	Not reported	Images	Dance categories: Ballet, hip-hop, pole, salsa, Samba, Bharatnatyam, Chaau, Dandya, Dhunuchi, Kathak, Kalbelia, and Kanipuri.	Sports, yoga, and dance (SYD) postures (including samba)
Chavoshi et al. (2014)	Three dancers: one teacher and two students.	Motion capture (MoCap) system recording object positions over time using reflective markers and infrared cameras	Basic movements of samba (the head, right finger, left finger, right toe, and left toe)	Not reported
Clarke et al. (2021)	83 female dance undergraduates	Real-time performance	Pirouettes	Ballet
Fang and Sun (2021)	Not reported	DanceSport video images (badminton sports video)	Positions of the head and feet	Sharpness data of the image (eight group)
Geukes et al. (2023)	33 perceivers	Video-records of 70 solo performances of a contemporary dance choreography	Choreography consisting of 6 × 8 counts, including walks, small and big jumps, dynamic leg elevations, pirouettes, balances, a split, and floor elements.	Ballet
Han et al. (2015)	20 Sport dancer	Real-time performance	The ankle, knee, spine, shoulder, and fingers.	Aerobic gymnastics, swimming, badminton, football (Soccer), control group
Kulis and Gajewski (2022)	Six high-level and six intermediate dance couples; six international adjudicators	Real-time performance	Basic Viennese Waltz figure: the “natural turn”	Not reported
Niewiadomski et al. (2019)	Seven athletes with various levels of experience and age; 14 karate experts	MoCap module: 3D positions of body joints from a motion-capture system; video module: video streams from one or more video cameras and/or RGB-D sensors; audio module: environmental or on-body nonverbal audio streams; physiological-signal module: data from physiological sensors	Two different katas (Heian Yondan and Bassai Dai)	Not reported
Ofori et al. (2021)	Fifteen healthy young participants	Participants dance to three songs of slow (120 beats per min, bpm), medium (130 bpm), and fast pace (138 bpm)	Right and left thighs, right and left shanks, and right and left foot joints	Silver-standard optoelectronic motion-capture system
Cui and Guo (2023)	Two professional DanceSport coaches and two well-known dancers	Scores assigned by comparing standard dance movements in teaching videos with the degree of overlap in trainees' dance-training videos	Modern dances: Waltz, Viennese Waltz, Tango, Foxtrot, and Quickstep; Latin dances: Rumba, Cha-Cha-Cha, Samba, Jive, and Paso Doble	Referee scoring and system scoring
Oliveira et al. (2012)	Two professional female dancers for dance recording and 15 subjects for subjective assessment	Recorded dances	Simple dance gestures in samba-no-pé style, dance gestures in the basic American Charleston style.	Dance sequences at four temporal resolutions: beat, half-beat, quarter-beat, and eighth-beat
Orlandi et al. (2020)	*N* = 41	24 dance video clips	Choreography duo movement sequences	Uniform dance (constant speed), varied dance (varied kinematics)
Prosen et al. (2013)	24 dance couples	Recorded video from the 2011 International DanceSport Federation competition	Turn of viennese waltz	Top-ranked 12 couples and lower-ranked 12 couples
Stancin and Tomazic (2021)	Two female dancers	Real-time performance	15 different solo jazz dance moves [(1) tackie annie; (2) fall of the log; (3) kicks; (4) half break; (5) struttin; (6) savoy kick; (7) 20′s charleston; (8) knee slaps; (9) fishtails; (10) apple jacks; (11) boogie back; (12) boogie forward; (13) crazy leg; (14) cross step; and (15) shorty george.]	Professional and recreational dancer
Sun and Wu (2023)	Not reported	Active motion-tracking mode of DanceSport video	Not reported	Not reported
Zhu et al. (2023)	25 subjects	Real-time performance in laboratory (wear in markers and IMUs)	Fitness dance movements, including certain zumba actions such as side jumps with arm swings and hip rotations.	Yoga, golf, swimming, and badminton group
Premelč et al. (2019)	18 judges, 12 adult dancing couples	Video of an international competition, the International Open 2012 in Ljubljana, Slovenia	Dance solo and two dances	Not reported

##### 3.3.2.1 Assessment methods and data overview

The reviewed studies used several types of data and validation procedures to test DanceSport assessment systems. These can be grouped into four broad categories: performance-based data, recorded visual data, pre-existing datasets, and interview- or feedback-based validation. Performance-based data were the common and included live or real-time performances, such as Viennese Waltz turns, Samba movements, pirouettes, dance tempo tasks, and laboratory-based movement trials. Recorded visual data were also widely used, including videos of correct and incorrect Tango performances, international competition videos, choreography clips, and sport-dance training videos (Gallo et al., 2022; Orlandi et al., 2020; Sun and Wu, 2023). A smaller number of studies relied on pre-existing datasets, including large motion or audio-visual datasets (Oh et al., 2014; Schuller et al., 2008) and image-based data (Bera et al., 2023). In addition, several studies incorporated interviews or feedback from experts and evaluated participants as part of the validation process (Beck et al., 2024; Oliveira et al., 2012; Premelč et al., 2019).

These data sources show that DanceSport assessment research has moved beyond purely judge-based scoring and now draws on video, motion-capture, sensor, image, and expert-feedback data. However, the evidence base remains heterogeneous. Some studies tested systems in relatively realistic competition or performance contexts, such as national championships, international competitions, or real-time dance performances, whereas others relied on laboratory tasks, pre-existing datasets, isolated body-joint data, or non-DanceSport proxy activities. This variation makes it difficult to compare the validity of different assessment systems directly.

##### 3.3.2.2 Participant types

Participant composition varied considerably across the reviewed studies. Some studies involved professional or high-level dancers, judges, trained performers, or adjudicators, whereas others included students, beginners, recreational dancers, or participants without formal dance experience. For example, Amoruso et al. (2014) included 80 participants and compared expert dancers, beginners, and non-dancers to examine performance-related expertise in Tango. Kulis and Gajewski (2022) involved six high-level and six intermediate dance couples together with six international adjudicators, allowing dance level and expert judgment to be considered within the same design. Premelč et al. (2019) included 18 judges and 12 adult dancing couples, providing a validation setting closer to formal DanceSport adjudication.

By contrast, several technology-oriented studies used smaller or more task-specific samples. Chavoshi et al. (2014), for instance, validated Samba movement capture with three dancers, while Stancin and Tomazic (2021) used two female dancers to test tempo detection in solo jazz movements. Other studies drew on participants from adjacent movement or sport backgrounds, such as ballet, contemporary dance, karate, fitness dance, yoga, golf, swimming, badminton, or general dance training contexts. These participant patterns indicate that validation samples were often selected to test whether a tool could capture or classify movement, rather than to evaluate its robustness across the full range of DanceSport performers, adjudicators, and competitive conditions.

##### 3.3.2.3 Dance movement coverage

The reviewed studies covered a wide range of movement tasks, but the scope of movement coverage remained uneven across studies. The tasks examined ranged from classical Tango steps, Samba basic movements, Viennese Waltz natural turns, pirouettes, solo jazz dance moves, dance gestures, and choreography-based sequences to isolated body-joint actions, posture images, gait dynamics, and fitness-dance movements. Some studies analyzed competition-related materials, such as Viennese Waltz turns in competition video or DanceSport performances from international events (Premelč et al., 2019; Prosen et al., 2013). Others examined simplified or segmented movement tasks designed for technical measurement, such as shoulder-joint angle estimation, balance tests, marker-based motion capture, or short recorded video clips.

This distribution shows that validation materials were more developed for isolated movement features than for complete DanceSport performance. Many studies focused on posture, turns, balance, timing, body-joint trajectories, or movement recognition, whereas fewer studies tested full routines, partner interaction, musical synchronization, expressive quality, or complete competition-level performance contexts. As a result, the movement materials used for validation differed substantially across studies, and relatively few designs approximated the complexity of formal DanceSport competition.

##### 3.3.2.4 Comparison logic and validation strength

Some studies strengthened validation through inter-group comparisons across skill levels, dance backgrounds, or reference methods. These included comparisons between expert dancers, beginners, and non-dancers (Amoruso et al., 2014), advanced and intermediate dancers (Kulis and Gajewski, 2022), professional and recreational dancers (Stancin and Tomazic, 2021), and higher- and lower-ranked competition couples (Prosen et al., 2013). Other studies compared DanceSport-related groups with participants from adjacent movement or sport backgrounds, such as gymnastics, swimming, badminton, football, yoga, golf, and karate (Han et al., 2015; Niewiadomski et al., 2019; Zhu et al., 2023). A smaller number of studies compared assessment approaches directly, such as referee scoring and system scoring (Cui and Guo, 2023), or modeled rater-based scoring structures within a formal competition setting (Anderlucci et al., 2021).

These comparison-based designs are important because they test whether assessment tools can distinguish expertise levels, movement qualities, scoring patterns, or methodological outputs. However, such validation was not consistently implemented across the reviewed literature. Many systems demonstrated that a movement feature could be detected, classified, estimated, or visualized, but fewer studies evaluated whether the resulting indicators improved judging reliability, aligned with formal adjudication criteria, or generalized to real competition settings. Overall, the validation evidence remains methodologically diverse and only partially connected to formal DanceSport scoring logic.

Overall, the Results show that DanceSport assessment research has moved from concerns with judging validity and performance testing toward technology-assisted measurement of movement, posture, rhythm, and dynamic features. However, the evidence base remains uneven across dance categories, assessment dimensions, and validation designs. Current studies are stronger in technical measurement than in establishing integrated, competition-level assessment validity.

## 4 Discussion

This review demonstrates that a Human-In-The-Loop systematic review framework is feasible for synthesizing DanceSport assessment research, despite not having been previously applied in this field. By combining exploratory LDA topic modeling with qualitative content analysis, the review identified not only the thematic organization of the field but also the methodological conditions under which assessment systems have been developed and validated. Four main findings emerged from this review and are discussed in the following sections.

### 4.1 Major research themes in DanceSport assessment research

With respect to RQ1, the findings show that the current thematic structure of DanceSport assessment research is strongly oriented toward technology-supported measurement. This pattern responds to the broader question of how dance performance can be assessed in ways that are both rigorous and publicly legitimate. The reviewed literature indicates that technology-assisted approaches have already become a major component of the field.

The exploratory LDA results showed that three of the four major themes were directly related to technical measurement, including 3D dance motion perception, dance posture and motion evaluation, and image analysis of dynamic dance performance. These themes focus on performance elements that can be captured and analyzed as movement data, such as posture, balance, rhythm, and spatial motion. The qualitative content analysis and human–computer interaction findings further support this pattern, showing widespread use of video analysis, motion capture, wearable sensors, and computational modeling. Taken together, these findings indicate that DanceSport assessment research is no longer organized only around judge-centered evaluation, but increasingly around the construction of measurable performance evidence.

At the same time, the persistence of validation- and judging-related themes indicates that expert adjudication remains central to DanceSport assessment. This is not surprising, because DanceSport evaluation is more subjective and multidimensional than many competitive sports: it involves not only measurable technical qualities, but also aesthetic judgments related to interpretation, expression, presentation, and appearance (Geukes et al., 2023). For this reason, a technology-oriented thematic structure does not imply the disappearance of judging systems. Rather, it suggests that current research is attempting to supplement, refine, and partially formalize adjudication by introducing more structured forms of performance evidence.

### 4.2 Development of research themes and the emerging assessment paradigm

With respect to RQ2, the stage-based topic modeling suggests that DanceSport assessment research has developed from concerns with judging standards and evaluation validity toward more technology-assisted forms of performance analysis. In the early stage, the central issue was how DanceSport performance should be judged and how scoring reliability could be stabilized in a field grounded in expert observation. This concern is consistent with the broader institutional importance of scoring in sport, where athletes' problems are often organized around technical outcomes such as scoring, ranking, and completing required routines (Hopsicker, 2015, p. 214). For DanceSport, however, scoring has never been a purely technical matter, because competitive judgment also carries implications for artistic value, institutional legitimacy, commercial visibility, and the sport's long-standing aspiration toward Olympic recognition (Picart, 2006, p. 84).

The later stages indicate a growing effort to transform DanceSport performance into analyzable data. Image analysis, posture datasets, motion capture, movement modeling, angle estimation, and rhythm-related information processing show that researchers increasingly approach performance as a measurable object. This trajectory resembles broader developments in aesthetic sports, where evaluation systems have attempted to coordinate technical objectivity with artistic judgment. As noted in the Introduction, figure skating provides a useful comparative reference; however, the reviewed DanceSport literature suggests that technical measurement, aesthetic interpretation, and competition-level adjudication remain insufficiently integrated. Therefore, the recent development of DanceSport assessment research should be understood as an emerging movement toward evidence-supported evaluation rather than as the establishment of a mature assessment framework.

At the same time, this trajectory should not be interpreted as straightforward progress toward neutrality. Technological objectivity is not the same as evaluative neutrality. Motion capture, image recognition, sensor systems, and machine-learning models may reduce some forms of perceptual inconsistency, but they also introduce new dependencies on datasets, movement segmentation, training samples, algorithmic assumptions, and the selection of measurable indicators. The experience of breaking within the Olympic context illustrates this tension: while sportification may enhance visibility and institutional recognition, standardized athletic criteria may also risk subordinating the aesthetic distinctiveness of dance performance (Li and Vexler, 2019). For DanceSport, this risk is particularly important because the field must balance technical precision with musicality, expressiveness, partner interaction, and stylistic interpretation.

The recent expansion stage therefore reflects methodological diversification rather than full system maturity. The field now captures more dimensions of performance than before, but it still lacks a stable framework that integrates technical precision, stylistic differentiation, and ecologically valid competition-level assessment. The emerging paradigm is best understood not as the replacement of expert adjudication by technology, but as a reconfiguration of assessment evidence: technical systems increasingly provide measurable performance data, while expert judgment remains necessary for interpreting aesthetic and competitive meaning.

### 4.3 Technologies and methodological approaches in DanceSport assessment

With respect to RQ3, the qualitative findings indicate that DanceSport assessment research has adopted diverse technological approaches, including visual and video analysis, motion capture and kinematic measurement, wearable and sensor-based data collection, computational recognition and modeling, as well as expert scoring and psychometric methods. These approaches broaden the evidential basis of assessment by making posture, balance, speed, turns, rhythm, joint movement, and coordination more observable and quantifiable. However, as DanceSport is an aesthetic sport, the value of these technologies lies not only in measuring movement accurately but also in supporting the interpretation of performance quality.

This distinction is critical because DanceSport assessment cannot be reduced to biomechanical or computational measurement alone. Situated at the intersection of sport and art, DanceSport derives value from both athletic performance and aesthetic experience (Hopsicker, 2015; Markula, 2018). While technical indicators capture aspects of movement execution, they do not fully explain musicality, partner interaction, expressive intent, stylistic character, or aesthetic impact. Existing systems therefore strengthen the technical foundation of assessment but remain limited in addressing the broader interplay among technique, athleticism, psychology, and aesthetics highlighted by Premelč et al. (2019) and Markula (2018).

Furthermore, aesthetic evaluation in DanceSport is shaped not only by movement quality but also by cultural and social contexts. Dai et al. (2024) showed that DanceSport performances are interpreted through culturally specific frameworks in which international technical standards intersect with local cultural, political, and gender norms. Judgments of performance quality may therefore reflect historically established standards, embodied practices, and culturally embedded expectations. Technically demanding elements such as standardized movement vocabularies, flexibility, turns, and lifts are often privileged because they align with dominant competitive discourses and internationally recognized ideals. At the same time, performers may negotiate cultural identities and gender expectations through choreography and role portrayal. These findings suggest that aesthetic judgment contains culturally contingent dimensions that are largely absent from current assessment technologies. Notably, none of the reviewed studies explicitly incorporated cultural variability into assessment models, highlighting an important gap in the literature.

A similar issue arises in the relationship between technological assessment and adjudication. Judge-based studies largely follow traditional adjudication frameworks and seek to improve scoring validity, reliability, and consistency. This remains essential because adjudicators must evaluate multiple couples simultaneously within large competition spaces and under significant time pressure, while maintaining attention and filtering irrelevant stimuli (Monleon et al., 2018). Fatigue, arousal, vigilance decline, and reduced cognitive resources can further influence decision-making (Niewiadomski et al., 2019). Technology can provide more stable and objective performance evidence, but it cannot replace the interpretive role of adjudicators in evaluating artistic and competitive significance.

Research on dance observation further suggests that aesthetic judgment emerges from complex perceptual, emotional, and cognitive processes. Christensen and Calvo-Merino (2013) argued that understanding the perceptual, affective, and cognitive mechanisms underlying movement observation, together with their neural correlates, is essential for explaining aesthetic experiences in dance. From this perspective, aesthetic evaluation depends not only on observable movement characteristics but also on how observers perceive, process, and emotionally respond to performance. Monleon et al. (2018) further proposed that insights from cognitive and neuroscientific research could help reduce subjective variability and cognitive limitations in adjudication. Despite this potential, none of the reviewed studies employed neurocognitive, neurophysiological, or observer-response approaches to investigate DanceSport assessment. This absence points to a promising research direction. Integrating cognitive neuroscience with performance analysis may help explain how judges form aesthetic judgments, identify sources of inconsistency, and strengthen theoretical links between measurable movement features and perceived performance quality.

Another challenge is that DanceSport is not a homogeneous movement domain. International Standard and Latin dances differ substantially in movement structure, posture, rhythm, partner interaction, and expressive conventions. Consequently, methods developed in one context may not transfer directly to another. This issue is particularly relevant because several reviewed studies drew on adjacent disciplines such as ballet, jazz, yoga, karate, and other movement forms. Although dance movement mechanics provide useful principles for analyzing flexibility, coordination, joint movement, balance, and bodily control (Honglian et al., 2021), and cross-domain comparisons can enrich movement-analysis research (Bera et al., 2023; Zenic et al., 2010), their applicability remains limited. Models trained to recognize posture or movement quality in one domain cannot automatically capture the stylistic, relational, cultural, and aesthetic demands specific to DanceSport.

Methodologically, future DanceSport assessment systems should move beyond simply incorporating additional sensors or algorithms. Greater attention should be given to clarifying how technical indicators relate to adjudication criteria and aesthetic dimensions of performance. In particular, assessment models need to explain how measurable variables such as angle, velocity, rhythm, balance, and movement regularity contribute to higher-order constructs including musicality, expressiveness, partner interaction, stylistic interpretation, cultural meaning, and overall performance quality. Without such an interpretive framework, technology-assisted assessment risks generating precise measurements with limited evaluative relevance. Future research would therefore benefit from integrating biomechanical, computational, psychological, cultural, and neuroscientific perspectives to develop more comprehensive assessment frameworks that better reflect the multidimensional nature of DanceSport performance.

### 4.4 Validation designs and the problem of assessment validity

With respect to RQ4, the reviewed studies show that existing DanceSport assessment systems have been validated through highly heterogeneous designs. Most studies reported some form of empirical testing, but they differed substantially in data sources, participant composition, movement tasks, and comparison structures. This heterogeneity is not merely a methodological detail; it directly affects whether the resulting systems can be compared, generalized, and interpreted as valid tools for DanceSport assessment.

A central issue concerns ecological validity. Earlier dance-perception studies often used edited stimuli to control experimental variables, such as reducing information richness, representing bodies through simplified lines, or obscuring facial and costume details (Christensen and Calvo-Merino, 2013; Cross et al., 2006). Such designs are valuable for isolating specific perceptual or technical dimensions, but they also move assessment away from the natural complexity of DanceSport performance. Formal DanceSport competition involves continuous routines, partner interaction, music synchronization, spatial navigation, multiple couples, time pressure, and adjudication under competitive conditions. Therefore, a system that performs well on isolated movements, short clips, laboratory tasks, or simplified visual materials may not necessarily support valid assessment in competition settings.

This tension reflects a broader methodological conflict between scientific control and performance naturalism. Highly controlled designs allow researchers to identify the effect of specific movement variables, but DanceSport assessment requires judgments of integrated performance quality. Geukes et al. (2023) offers a useful example of a more ecologically sensitive approach by using a lens model to examine how perceivers evaluated dance through multiple naturally varying cues, including skill level, physical characteristics, and personality, while controlling key factors such as choreography, music, and dance style. This type of design suggests that richer data sources can strengthen assessment validity by linking measurable performance features with perceptual and evaluative outcomes.

Participant heterogeneity further complicates validation. The reviewed studies included professional dancers, judges, trained performers, students, beginners, non-dancers, and participants from adjacent sport backgrounds. This diversity can be useful for testing whether systems distinguish expertise levels or general movement qualities, but it also limits direct comparison across studies. Results obtained from small samples, non-DanceSport participants, or adjacent movement domains cannot be automatically generalized to competitive DanceSport. Moreover, few studies incorporated multiple stakeholder groups, such as dancers, judges, coaches, and audience members, within the same validation design. This limits understanding of how different evaluative perspectives converge or diverge.

The same problem appears in the use of validation data. Video, image, sensor, motion-capture, physiological, interview, judge-rating, and audience-response data each capture different aspects of performance. Quantitative movement trajectories and physiological signals can provide evidence of technical execution, whereas interviews, expert judgments, and audience responses can better represent aesthetic perception and evaluative meaning. A comprehensive validation framework should therefore not rely on a single data type, but should integrate technical, perceptual, and interpretive evidence. Such integration is especially important for future AI-assisted assessment systems, because model performance depends heavily on the quality, diversity, and relevance of the datasets used for training and testing.

The main consequence of current validation heterogeneity is that the field accumulates technical demonstrations rather than comparable evidence for assessment validity. Many studies show that a movement feature can be detected, classified, estimated, or visualized, but fewer studies test whether these indicators improve judging reliability, align with formal adjudication criteria, or generalize to full competition contexts. Future validation should therefore prioritize competition-level datasets, complete routines, expert-rating benchmarks, cross-category comparisons between International Standard and Latin dances, and multi-stakeholder evaluation designs. Although the field does not yet have a single “correct” validation model, the direction is clear: valid DanceSport assessment requires linking technical measurement with naturalistic performance conditions and human evaluative judgment.

## 5 Conclusion and future research

This study conducted a systematic review enhanced by Human-In-The-Loop topic modeling to examine the thematic structure, methodological characteristics, and validation designs of DanceSport assessment research. The findings indicate that the field has made significant progress through technology-assisted and data-driven approaches, particularly in motion analysis, posture recognition, sensor-based measurement, computational modeling, and expert-scoring refinement. However, future progress in DanceSport assessment will depend less on the accumulation of isolated indicators and more on the development of frameworks that can relate technical measurement to the broader logic of performance evaluation. For DanceSport, this means that assessment systems should no longer optimize only for isolated metrics such as step accuracy or pose recognition, but should be validated under competition-representative conditions involving continuous choreography, partner interaction, music synchronization, aesthetic presentation, and judging constraints.

Future research should therefore move toward a comparable evidence ecosystem supported by shared datasets, transparent validation protocols, and reproducible reporting practices. More specifically, the field needs cross-dance standard datasets, comparative studies between International Standard Dance and International Latin Dance, validation based on complete competition routines, and criterion-validity testing between technical indicators and expert scores. Future assessment frameworks should also involve judges, coaches, and athletes, so that technical indicators can be interpreted in relation to adjudication criteria, training relevance, and performance practice. Increasingly, research also highlights the importance of human factors, interpretable models, and transparent quality-control pipelines, rather than relying solely on black-box predictive accuracy. In this context, future systems should be conceptualized as structured evidence generators that support human judgment by providing traceable, interpretable, and comparable performance information, rather than producing decontextualized final scores. If these directions are addressed, the refinement of assessment systems may not only improve evaluation practices but also enable DanceSport to evolve toward more diversified forms of competition, broader institutional recognition, and expanded visibility on international platforms.

## 6 Limitations

Although this study sought to provide a comprehensive overview of DanceSport assessment research through a systematic review combined with LDA topic modeling, several limitations should be acknowledged. First, although the literature search followed a transparent and systematic procedure, it was limited to the Web of Science Core Collection. As a result, relevant studies indexed in other databases may not have been captured, potentially affecting the completeness of the evidence base. Second, although the review synthesized all eligible studies identified through the screening process, only 24 studies met the inclusion criteria. This reflects the emerging nature of the field but also limits the breadth of evidence and introduces heterogeneity in research contexts, assessment methods, and validation approaches. Third, although LDA provided a useful exploratory overview of research themes, the topic model was based on a relatively small corpus of 24 abstracts rather than full-text articles. Consequently, some methodological details and contextual information may not have been fully represented, and the identified topics should be interpreted as indicative rather than definitive. To reduce this limitation, topic-modeling results were interpreted alongside full-text qualitative analysis. Finally, although qualitative coding and interpretation were conducted systematically and guided by established principles of thematic analysis (Nowell et al., 2017), the interpretation of themes inevitably involved researcher judgment. In addition, the quality appraisal focused on reporting transparency rather than formal risk-of-bias assessment, and the considerable variation in validation strategies across studies limits direct comparison of assessment approaches. Future research would benefit from broader database coverage, larger evidence bases, and more standardized validation frameworks for DanceSport assessment.

## Data Availability

The original contributions presented in the study are included in the article/Supplementary material, further inquiries can be directed to the corresponding author.
