# Supplementary material for: Mapping assessment practices in DanceSport: a Human-In-The-Loop systematic review
Source: Front Psychol. 2026 Jul 22;17:1847089. doi: 10.3389/fpsyg.2026.1847089 (PMC13437966; doi:10.3389/fpsyg.2026.1847089)
Supplement: Supplementary file 1 [file Data_Sheet_1.pdf]

Supplementary Material

1 Supplementary Tables

Supplementary Table S1. Database, search settings, and screening parameters

**Item**	**Information to report**
Database	Web of Science Core Collection (WOS)
Indexes	SCI-EXPANDED, SSCI, A&HCI, ESCI
Search date	August 1, 2024
Search field	Topic
Time span	2000 to 2024
Language	English only
Document type	Article only, excluding reviews, proceedings, conference abstracts, book chapters, non-peer-reviewed items
Exact search strings	(TS=(sport dance)) AND TS=(assessment)<br>(TS=("sport dance")) AND TS=(assessment)<br>(TS=("sport dance")) AND TS=(performance)<br>(TS=(Dancesport)) AND TS=(assessment)<br>(TS=(Dancesport)) AND TS=(performance)<br>(TS=("competitive ballroom dancing")) AND TS=(assessment)<br>(TS=("competitive ballroom dancing")) AND TS=(performance)<br>(TS=(Waltz)) AND TS=(assessment)<br>(TS=(Waltz)) AND TS=(performance)<br>(TS=(Tango)) AND TS=(assessment)<br>(TS=(Tango)) AND TS=(performance)<br>(TS=("Viennese Waltz")) AND TS=(assessment)<br>(TS=("Viennese Waltz")) AND TS=(performance)<br>(TS=("Slow Foxtrot")) AND TS=(assessment)<br>(TS=("Slow Foxtrot")) AND TS=(performance)<br>(TS=(Quickstep)) AND TS=(assessment)<br>(TS=(Quickstep)) AND TS=(performance)<br>(TS=(Samba)) AND TS=(assessment)<br>(TS=(Samba)) AND TS=(performance)<br>(TS=("Cha-Cha-Cha")) AND TS=(assessment)<br>(TS=("Cha-Cha-Cha")) AND TS=(performance)<br>(TS=(Rumba)) AND TS=(assessment)<br>(TS=(Rumba)) AND TS=(performance)<br>(TS=("Paso Doble")) AND TS=(assessment)<br>(TS=("Paso Doble")) AND TS=(performance)<br>(TS=(Jive)) AND TS=(assessment)<br>(TS=(Jive)) AND TS=(performance)
Duplicate removal	Software (Zotero) /manual verification
Screening tool	Screening tool

Final records	1,957

Supplementary Table S2. Search keywords and retrieval outputs

Search Number	Keywords		Results (initial included)
1	sport dance	assessment	328(23)
2		performance	635(103)
3	Dancesport	assessment	10(7)
4		performance	32(16)
5	competitive ballroom dancing	assessment	2(1)
6		performance	20(5)
Standard dances			
7	Waltz	assessment	32(1)
8		performance	122(16)
9	Tango	assessment	134(0)
10		performance	343(17)
11	Viennese Waltz	assessment	1(1)
12		performance	7(1)
13	Slow Foxtrot	assessment	0
14		performance	0
15	Quickstep	assessment	1(0)
16		performance	24(1)
Latin-American dances			
17	Samba	assessment	55(1)
18		performance	140(12)
19	Cha-Cha-Cha	assessment	0
20		performance	2(2)
21	Rumba	assessment	4(1)
22		performance	16(3)
23	Paso Doble	assessment	2(0)
24		performance	3(0)
25	Jive	assessment	10
26		performance	33(1)
Total			1957(192)

Supplementary Table S3. Text preprocessing rules and lexical normalization procedures

Step	Procedure	Rule / Example
**Text field construction**	Abstracts of the included studies	The abstracts of the final 24 included studies were used as the textual basis for exploratory LDA topic modeling.
**Removal of non-relevant text**	Manual and rule-based cleaning	Non-English items, numerical tokens, punctuation, publisher names, copyright notices, and end-of-abstract publication information were removed where applicable.
**Lowercasing**	All tokens lowercased	“DanceSport” → “dancesport”; “Performance” → “performance”.

**Tokenization**	NLTK word tokenization	Abstract texts were segmented into word-level tokens.
**Stopword removal**	NLTK English stopwords plus customized stopword list	Standard English stopwords were combined with corpus-specific low-semantic-value words, such as “study,” “result,” “method,” and “paper,” where applicable.
**Part-of-speech filtering**	NLTK POS tagging	Verbs and procedural noise were removed; substantively meaningful non-stopword tokens and domain-relevant terms were retained.
**Domain vocabulary preservation**	Customized vocabulary list	Domain-specific terms were retained when they appeared in the customized vocabulary list.
**Synonym normalization**	Customized synonym mapping	Equivalent or closely related terms were normalized into unified forms, such as “ballroom dance,” “sport dance,” and “dancesport,” where applicable.
**Lemmatization**	Not applied as an independent preprocessing step	WordNet-based lemmatization was not used as a separate operation in the final preprocessing pipeline.
**Phrase handling**	Not automatically detected	Bigrams or multi-word expressions were not extracted through an automatic phrase-detection model; domain expressions were handled through vocabulary preservation and synonym normalization where applicable.
**Document-frequency filtering**	Not applied as an independent threshold	No separate minimum or maximum document-frequency threshold was applied in the final preprocessing pipeline.
**Salient term identification**	TF-IDF-assisted lexical inspection	TF-IDF was calculated to support lexical inspection and topic interpretation.
**Output for LDA**	Preprocessed token lists, dictionary, and bag-of-words corpus	Cleaned token lists were converted into a Gensim dictionary and bag-of-words corpus for LDA modeling.

Supplementary Table S4. LDA model settings and reproducibility information

**Parameter**	**Setting**
**Software**	Python; Gensim
**Corpus**	Abstracts of the 24 included studies
**Input representation**	Bag-of-words corpus
**Models estimated**	Overall corpus; Stage 1 corpus; Stage 2 corpus; Stage 3 corpus
**Candidate topic numbers**	K = 1–20
**Topic-number selection criteria**	Perplexity, c_v coherence, and researcher interpretability
**Passes**	20
**Random seed**	42
**Alpha**	auto
**Eta / beta**	auto

**Topic labeling**	Topic labels were assigned through researcher interpretation of high-probability keywords and representative study content within the Human-In-The-Loop review framework.

Supplementary Table S5. Topic similarity matrix across temporal stages (Links)

Source topic	Target topic	Link value / cosine similarity	Shared top keywords	Interpretation
Stage 1 → Stage 2				
Stage 1 Topic 1	Stage 2 Topic 1	0.2	sports; dance	Early performance/training-oriented terms connect to later image/posture-recognition topics through the shared DanceSport vocabulary.
Stage 1 Topic 1	Stage 2 Topic 3	0.2	dance; system	Early system-related performance testing connects to later motion-capture and system-validity topics.
Stage 1 Topic 2	Stage 2 Topic 3	0.1	validity	Early judging-validity concerns connect weakly to later system-validity and motion-capture evaluation.
Stage 2 → Stage 3				
Stage 2 Topic 1	Stage 3 Topic 1	0.1	performance	Image/posture-recognition themes connect weakly to later expertise and performance themes.
Stage 2 Topic 1	Stage 3 Topic 2	0.1	performance	Image/posture-recognition themes connect to motion-estimation evaluation through performance-related vocabulary.
Stage 2 Topic 1	Stage 3 Topic 3	0.1	dance	Image/posture-recognition themes connect weakly to later movement-feature and aesthetic-system topics.
Stage 2 Topic 1	Stage 3 Topic 4	0.1	dance	Image/posture-recognition themes connect weakly to judgment-dynamics and technique-related topics.

Stage 2 Topic 1	Stage 3 Topic 5	0.1	dance	Image/posture-recognition themes connect weakly to ballroom style and music-information topics.
Stage 2 Topic 2	Stage 3 Topic 1	0.1	performance	Couple movement themes connect weakly to later expertise/performance themes.
Stage 2 Topic 2	Stage 3 Topic 2	0.2	movement; performance	Couple movement themes show a stronger connection with motion-estimation and performance-evaluation themes.
Stage 2 Topic 2	Stage 3 Topic 3	0.1	movement	Couple movement themes connect to later movement-feature analysis.
Stage 2 Topic 3	Stage 3 Topic 1	0.1	performance	Motion-capture validity themes connect weakly to later expertise/performance themes.
Stage 2 Topic 3	Stage 3 Topic 2	0.1	performance	Motion-capture validity themes connect weakly to motion-estimation evaluation.
Stage 2 Topic 3	Stage 3 Topic 3	0.2	dance; system	Motion-capture and system-validity themes show a stronger connection with later movement-feature and system-oriented topics.
Stage 2 Topic 3	Stage 3 Topic 4	0.1	dance	Motion-capture validity themes connect weakly to judgment-dynamics topics.
Stage 2 Topic 3	Stage 3 Topic 5	0.1	dance	Motion-capture validity themes connect weakly to ballroom style and rhythm/music topics.

Note. Each topic was represented by its top ten LDA keywords. Cosine similarity was calculated between binary word-presence vectors of adjacent-stage topic pairs. The visualization threshold was set at cosine similarity ≥ 0.1, corresponding to at least one shared top keyword. Links with similarity = 0 were omitted. Link thickness in the Sankey diagram was proportional to the cosine similarity score.

Supplementary Table S6. Coding framework for qualitative content analysis

Domain	Coding variable	Code / Category	Operational definition	Coding example
	Category of dance	Standard Dance	Study examines one or more International	Waltz; Tango; Viennese Waltz

**Assessment system profile**			Standard dances or Standard-style DanceSport contexts.	
		Latin Dance	Study examines one or more International Latin-American dances or Latin-style DanceSport contexts.	Samba; Rumba; Cha-Cha-Cha
		Mixed / general DanceSport	Study examines DanceSport broadly or includes both Standard and Latin categories.	DanceSport competition; ballroom dance performance
		Methodologically transferable	Study is not explicitly labeled as DanceSport but provides transferable assessment logic, movement-analysis procedure, or validation design.	Dance posture recognition using expert-labeled movement data
	Assessment method	Judge-based evaluation	Assessment relies primarily on human judges, experts, referees, or rating scales.	Expert score comparison; judge performance analysis
		Software-assisted evaluation	Assessment uses computational tools, algorithms, software platforms, or visual analytics to support evaluation.	Image-based posture recognition; web-based choreography assessment
		Hardware-supported / multimodal evaluation	Assessment combines software with sensors, cameras, motion capture, wearable devices, or other hardware systems.	Motion-capture-based movement assessment
	Assessment targets	Performance quality	Assessment addresses overall performance level, scoring quality, competitive success, or expertise.	Expert-rated performance quality; competition-level comparison
		Movement / posture	Assessment focuses on body position, movement accuracy, posture, joint angle,	Angle-based posture evaluation; balance test

			balance, or kinematic quality.	
		Choreography / formation	Assessment focuses on choreography structure, formation planning, spatial organization, or routine design.	Formation assessment in choreography visualization
	Assessment technology	Expert rating / psychometric framework	Assessment uses expert scoring, judge ratings, rating-scale analysis, or psychometric modeling.	Many-facet Rasch analysis of judges' scores
		Video / image-based analysis	Assessment uses images, videos, pose estimation, computer vision, or deep-learning recognition.	CNN-based dance posture recognition
		Motion capture / kinematic analysis	Assessment uses motion capture, inertial sensors, biomechanical features, or kinematic measurements.	3D motion tracking; joint-angle analysis
		Visualization / software system	Assessment uses software platforms or visualization systems to support analysis, planning, or feedback.	ChoreoVis; MotionPerfection
**Validation experiment design**	Participants	Dancers / athletes	Study includes dancers, DanceSport athletes, or participants with dance training.	Expert dancers; beginner dancers; undergraduate dancers
		Judges / experts / coaches	Study includes judges, referees, coaches, or domain experts as evaluators or participants.	DanceSport judges; expert raters
		Non-expert / control participants	Study includes non-dancers, novices, naïve observers, or general participants.	Naïve observers; novice participants
	Evaluation data sources	Expert scores / ratings	Validation or assessment data are derived from judge	Expert score used as reference standard

		scores, expert ratings, or referee evaluations.	
	Recorded visual data	Validation or assessment data are based on video, image, or recorded performance material.	Recorded dance performance videos
	Sensor / motion data	Validation or assessment data are derived from motion capture, inertial sensors, physiological signals, or kinematic records.	MoCap trajectories; IMU data
	Dataset / software output	Validation or assessment data come from existing datasets, algorithmic outputs, or software-generated features.	SYD posture dataset; pose-recognition output
Dance movement	Isolated movement / posture	Study uses a single movement, posture, balance task, step, or short movement segment.	Single turn; posture image; pirouette test
	Dance sequence / routine segment	Study uses a short dance sequence, selected routine segment, or repeated movement pattern.	Basic Samba movement sequence
	Competition-like routine / performance context	Study uses extended choreography, partnered performance, real-time performance, or competition-related material.	National competition score data; full performance recording
Comparison between groups	Expertise / level comparison	Study compares participants or performances across expertise, skill, or competition levels.	Expert dancers vs beginners
	Method / system comparison	Study compares assessment methods, algorithmic outputs, or system performance.	Algorithmic score compared with expert rating

Condition / task comparison	Study compares different movement conditions, task types, or evaluation settings.	Correct vs incorrect performance videos

Supplementary Table S7. Simplified methodological quality appraisal of included studies

Study	Study type	Sample characteristics	Validation design	Comparison group	Data source clarity	Indicator definition	Method reproducibility	Total score	Overall reporting quality
**Author year**	Yes/Partial/No	Yes/Partial/No	Yes/Partial/No	Yes/Partial/No	Yes/Partial/No	Yes/Partial/No	Yes/Partial/No		High/Moderate/Low

Notes: Yes = 2, Partial = 1, No = 0. Total score range = 0–12. High reporting quality = 10–12; moderate = 6–9; low = 0–5. This appraisal was used to contextualize evidence transparency and comparability, not to exclude studies.

2 Supplementary Figures

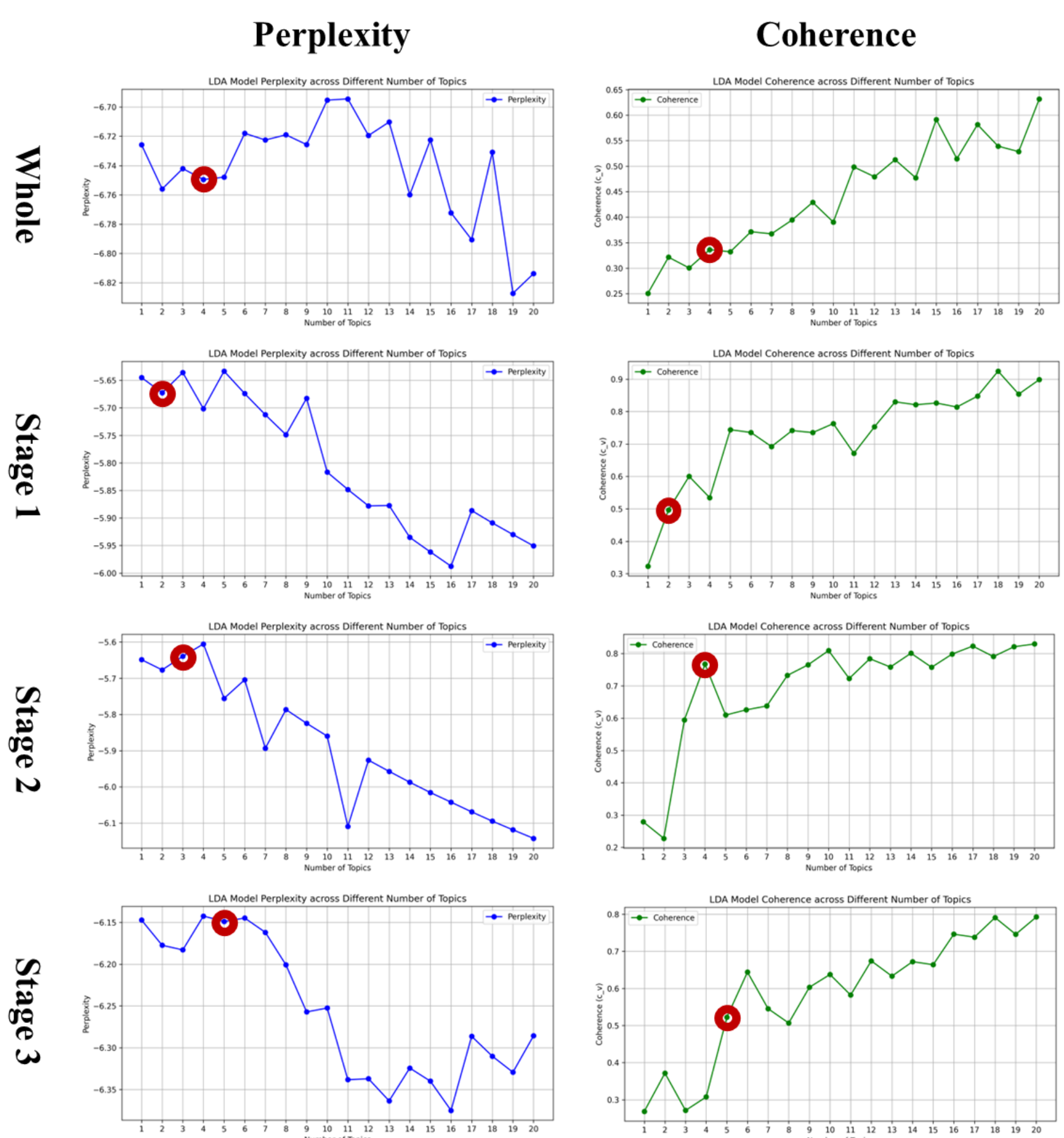

Supplementary Figure 1. Perplexity and coherence results for determining the number of topics in the overall corpus and stage-specific corpora.

3 Supplementary Formula

Supplementary Formula S1. Perplexity

$$\mathrm{p}erplexity(D) = \exp\left\{ -\frac{\sum_{d=1}^{M} \log p(w_d)}{\sum_{d=1}^{M} N_d} \right\}$$

where D is the document set, exp{} is an exponential function with the natural logarithm e as the base, $p(w_d)$ is the generation probability of document d, N_d represents the lexical length of document d, and M is the number of documents.

Supplementary Formula S2. Coherence

$$Coherence(V) = \sum_{(V_i, V_j \in V)} score\left(V_i, V_j, \in\right)$$

Here, V is a set of words describing the topic, and ϱ returns a smoothing factor for the real number to guarantee the score.

Supplementary Formula S3. Topic intensity

$$T_k = \frac{\sum_{d=1}^{M} \theta_k^d}{M}$$

Here, T_k is the intensity value of the topic k, and θ_d^k is the probability of the topic k appearing in the document d.

Supplementary Formula S4. Cosine similarity for thematic transition

$$\cos\text{inesimilarity}\left(A, B\right) = \cos\left(\theta\right) = \frac{A \cdot B}{AB} = \frac{\sum_{i=1}^{n} A_i B_i}{\sqrt{\sum_{i=1}^{n} A_i^2}\sqrt{\sum_{i=1}^{n} B_i^2}}$$

Where A and B are binary word-presence vectors constructed from the top ten keywords of two adjacent-stage topics. Here, Ai and Bi are vector representations of the two topics A and B.
